# Abnormal Sense of Agency in Patients with Schizophrenia: Evidence from Bimanual Coupling Paradigm

**DOI:** 10.3389/fnbeh.2016.00043

**Published:** 2016-03-09

**Authors:** Francesca Garbarini, Angela Mastropasqua, Monica Sigaudo, Marco Rabuffetti, Alessandro Piedimonte, Lorenzo Pia, Paola Rocca

**Affiliations:** ^1^SAMBA (SpAtial, Motor and Bodily Awareness) Research Group, Department of Psychology, University of TorinoTorino, Italy; ^2^Department of Neuroscience, Psychiatric Section, University of Turin, SSCVDTurin, Italy; ^3^Department of Biomedical Technology, IRCCS Don Carlo Gnocchi FoundationMilan, Italy; ^4^Neuroscience Institute of Turin (NIT), University of TurinTurin, Italy

**Keywords:** schizophrenia, positive symptoms, sense of body-ownership, sense of agency, bimanual coupling effect

## Abstract

A fruitful approach to the understanding the human awareness of action is the study of those pathologies in which some aspects of it are altered. Previous evidences showed that patients with schizophrenia tend to attribute someone else’ actions to their own, as internally, rather than externally, generated. Here, we asked whether schizophrenics have an “excessive” sense of agency, while observing others’ movements. We took advantage from the circles-lines task, known to show bimanual interferences. Twenty schizophrenics and 20 age-matched healthy controls were administered: (a) the bimanual version of the task: drawing lines with one hand and circles with the other; and (b) a modified version: drawing lines while observing the examiner drawing circles. In the bimanual version, patients and controls showed a comparable interference effect. In the observation version, schizophrenics, compared to controls, showed a significantly greater interference effect of the examiners’ hand drawing circles on the own hand drawing lines. This effect was significantly correlated to the strength of the positive symptoms (hallucinations and delusions) and to the alteration of the sense of agency, reported during the task. These findings suggest that an altered sense of agency, as shown by schizophrenics, can induce objective consequences on the motor system.

## Introduction

Despite the subjective experience of willed actions having a unitary nature, converging evidence in cognitive neuroscience have clearly pinpointed that conscious awareness of actions has a rich and multifaceted signature, and can be dissociated in different components. To this respect, a fundamental approach to the understanding of human awareness of action is the study of those pathological conditions, such as schizophrenia, in which some aspects of it are altered.

Schizophrenia is a well-known widespread mental disease characterized by a variety of signs and symptoms, which are differentially expressed across patients and through the course of the illness. Symptoms include characteristic distortions of thinking and perception, cognitive impairments, motor abnormalities, avolition and apathy, difficulties in communication, and restricted affective expression. These abnormalities are generally classified into positive, negative, cognitive, disorganization, mood, and motor symptom dimensions (Tandon et al., 2009). Within positive symptoms dimension, Schneider conceptualized First Rank Symptoms which are self-disturbances including passivity experiences or delusions of controls, in which the individual has the experience of the mind or body being under the influence or control of some kind of external force or agency (Schneider, 1959). This altered conscious awareness of action has been explained within the well-established forward model of motor control (Wolpert et al., 1995; Blakemore and Frith, 2003). The model states that when motor predictions match sensory consequences, the movement is perceived as one’s own. On the contrary, when there is a mismatch, the sense of being the agent of that action (i.e., sense of agency) decreases. According to this model, patients with schizophrenia are unable to create predictions of willed actions and, hence, experience movements as generated by external forces (Frith, 1992; Frith et al., 2000a,b; Blakemore et al., 2002; Lindner et al., 2005).

Such interpretation of a reduced sense of agency based on clinical symptoms, however, cannot easily explain those evidences showing that, in some experimental contexts, schizophrenics show, on the opposite, a hyper-sense of agency. Within this line of research, a first approach consists in asking participants to explicitly indicate how much they sense a feeling of control and of authorship over an externally generated action they can see on a screen. Previous literature employed these kinds of tasks in patients with schizophrenia, introducing, with a variety of techniques, temporal or spatial distortion of the visual feedbacks (i.e., body movements). Essentially, those studies showed that patients are inaccurate and tend to attribute what is seen to them, a sort of over-attribution of agency (Daprati et al., 1997; Franck et al., 2001; Synofzik et al., 2010). The second experimental approach to the study of agency in schizophrenics, involves implicit measures as, for instance, the temporal binding paradigm known to show in healthy participants that voluntary action and its sensory consequences bind each other in time (Haggard and Eimer, 1999; Haggard et al., 2002). The studies which administered this paradigm to patients affected by schizophrenia (Haggard et al., 2003; Voss et al., 2010) showed that they have stronger binding effects respect to the healthy population. In other words, their altered sense of agency consists in perceiving their own actions as having exaggerated causal effects on the external world.

In the present study, we proposed a new approach for studying the sense of agency in schizophrenia, asking whether, in these patients, an altered sense of agency may have consequences on the patients’ motor parameters. This working hypothesis was based on previous data recently obtained on neurological patients showing a monothematic delusion of body ownership (Garbarini and Pia, 2013; Garbarini et al., 2013b, 2014, 2015a; Pia et al., 2013). Specifically, whenever someone else’s hand is located in a 1st person perspective and in a body-congruent position, these patients falsely misattribute to themselves this latter hand as well as its movements. In other words, this means that when the sense of body ownership is altered, so that the alien hand is embodied, the sense of agency is altered as well. Moreover, this altered sense of agency is so deeply embedded within patients’ motor system as to affect even their motor performance. To study this delusion of body ownership, Garbarini et al. (2014) employed a modified version of circles-lines task known to show bimanual interferences in healthy subjects. When people have to draw lines with one hand while drawing circles with the other hand, each movement interferes with the other one and both trajectories tend to become ovals (curved lines or line-like circles); i.e., bimanual coupling effect (Franz, 1997; Garbarini et al., 2012, 2013a, 2015b; Piedimonte et al., 2014). Patients with delusion had to draw lines with their intact hand while watching an alien hand (the examiner’s one) performing circles (Garbarini et al., 2013b). When the alien hand drew circles, patients misidentified the hand as their own (i.e., altered sense of body ownership), misattributed its movements to themselves (i.e., altered sense of agency) and displayed a clear coupling effect (comparable to that found in normal individuals actually performing the bimanual task). It is important to note that, in the same condition, neither healthy controls nor hemiplegic patients without embodiment showed any coupling effect. This suggests that simply looking at a hand drawing circles is not sufficient to induce line ovalization.

Based on this previous evidence, in the present study we reasoned that this modified version of the circles-lines task (drawing lines while observing the examiner’s hand drawing circles) would have allowed us to make clear predictions respect to our main question in patients with schizophrenia. Does, in those patients, an altered sense of agency over the observed movement induces objective consequences on the patients’ motor performance? If patients with schizophrenia, particularly those with first-rank symptoms such as hallucinations and delusions (Daprati et al., 1997; Voss et al., 2010), have an “excessive” sense of agency, they would be more likely to perceive externally generated circles (drown by the examiner) as internally generated, as if they had to perform the bimanual task with both hands. Therefore, compared to controls, they should show a greater interference effect of someone else’s hand drawing circles on the own hand drawing lines.

## Materials and Methods

### Participants

The study has been conducted at the Department of Neuroscience, University of Turin, Struttura Semplice di Coordinamento a Valenza Dipartimentale (SSCVD) and at the Psychiatric Emergency Service (PES), Department of Neuroscience and Mental Health, A.O. Città della Salute e della Scienza di Torino—Presidio Molinette, Turin, during the period between January and June 2015.

For the study 40 participants were recruited, 20 patients with schizophrenia (10 females/10 males; mean age = 46.7, SD = 14.7) and 20 age-matched healthy controls (10 females/10 males; mean age = 45.2; SD = 12.4). All participants were right-handed according to the Edinburgh Inventory (Oldfield, 1971). Within the patients’ group, 10 acute inpatients, were recruited from PES; 10 stable outpatients, were recruited from SSCVD.

Patients all fulfilled formal Diagnostic and Statistical Manual of Mental Disorders (DSM-IV-TR; American Psychiatric Association, 2000) diagnostic criteria for schizophrenia. The diagnosis of schizophrenia was determined by treating psychiatrists and was confirmed by two expert clinicians (P.R., M.S.) using the Structured Clinical Interview for DSM-IV disorders (SCID; First, 2015). Patients’ demographic and clinical features are shown in Table 1. Subjects were excluded if they had a current disorder other than schizophrenia, a current or past co-diagnosis of autistic disorder or another pervasive developmental disorder, a history of severe head injury (coma ≥48 h), or a diagnosis of a psychiatric disorder due to a general medical condition. All the patients were receiving antipsychotic medication at the time of assessment. All the patients included in the study were treated with second-generation antipsychotics (SGAs; Table 2). None of the patients were receiving anticholinergic drugs at the time of assessment. In order to evaluate and exclude the effects of drug-induced EPS, patients were evaluated with the Modified Simpson-Angus Scale (MSAS; Simpson and Angus, 1970). The mean rating (± SD) of MSAS in our sample of patients was 2.6 ± 3.61, showing absence or minimal degree of movement disorder.

**Table 1:** **Patients’ demographic (a) and clinical (b) features**.

	Patients	Acute patients	Stable patients	Controls
(a) Demographic characteristics
Sample (N)	20	10	10	20
Gender	10 M; 10 F	6 M; 4 F	4 M; 6 F	10 M; 10 F
Age (years)	46.7 ± 14.7	49 ± 12.6	44.4 ± 16.9	45.2 ± 12.4
Education (years)	12 ± 3.9	11.3 ± 3.7	12.6 ± 4.1	13 ± 3.4
(b) Psychiatric assessment
Onset of schizophrenia (years)	26.4 ± 12.3	28.8 ± 16.1	23.9 ± 6.7	–
Duration of illness (years)	20.3 ± 13.6	20.2 ± 8	20.4 ± 18.1	–
CPZ equivalent (mg/die)	551.8 ± 464.5	707.4 ± 536.6	396.2 ± 340.7	–
SAPS global*	31.3 ± 27.4	51.4 ± 24.7	11.2 ± 8.9	–
(a) Hallucinations*	5.3 ± 7.9	9.3 ± 9.4	1.2 ± 2.7	–
(b) Delusions*	11.2 ± 10.8	19.7 ± 8.5	2.6 ± 3.7	–
(c) Bizarre Behavior*	5.3 ± 4.1	7.6 ± 2.9	2.9 ± 3.7	–
(d) Positive Formal Thought Disorder*	9.7 ± 8	14.8 ± 7.1	4.5 ± 5.3	–
SANS global	51.3 ± 23.7	59.8 ± 17	42.7 ± 27.1	–
(a) Affective Flattening or Blunting	15.4 ± 6.6	17 ± 4.3	13.7 ± 8.1	–
(b) Alogia	9 ± 5.8	9.9 ± 4.6	8 ± 6.9	–
(c) Avolition—Apathy*	8.4 ± 5.1	10.7 ± 3.8	6 ± 5.3	–
(d) Anhedonia—Asociality	12.5 ± 6.3	13.8 ± 5.4	11.1 ± 7.2	–
(e) Attention*	6.2 ± 3.9	8.4 ± 2.5	3.9 ± 3.8	–

*Indicate a significant difference (p < 0.05, Kruskal-Wallis non-parametric test) between acute and stable patients.

**Table 2:** **Patients drug intake**.

		Daily dose	
*N* patients	Drug (active principle)	Range (mg)	Mean (mg)
6	Aripiprazole	10–30	20
2	Clozapine	400–600	500
3	Olanzapine	2.5–20; 10–20	15
4	Paliperidone	9–12	11.25
2	Quetiapine	200–800	500
6	Risperidone	1–5	3
1	Ziprasidone	120	120

Twenty healthy control subjects with no history of neurological or psychiatric disease volunteered for this study. The two groups, patients with schizophrenia and healthy control, were matched for age, sex and handedness. Written informed consent was obtained from all subjects after a complete description of the study. The study was performed in accordance to the ethical standards of the Declaration of Helsinki and was approved by our Local Research Ethics Commitee (LREC; AOU Città della Salute e della Scienza, Turin, Italy).

### Psychiatric Assessment

Current levels of psychopathological symptoms were assessed using the Scale for the Assessment of Negative Symptoms (SANS; Andreasen, 1984a) and the Scale for the Assessment of Positive Symptoms (SAPS; Andreasen, 1984b). We decided to use SAPS and SANS for the assessment of psychopathology because we decided to focus on positive and negative symptoms of schizophrenia. Furthermore, this type of choice would allow us to a better comparison with previous results in the literature.

The SANS measures negative symptoms and consists of 22 items divided into five subscales (Affective Flattening or Blunting, Alogia, Avolition-Apathy, Anhedonia-Asociality, and Attention). A global score for each subscale intended to summarize all of the symptoms within a subscale category is also included. A semi-structured interview is used to make some of the item ratings, with additional ratings based on direct behavioral observation.

The SAPS consists of 34 items divided into four positive symptom subscales: hallucinations, delusions, bizarre behavior, and positive formal thought disorder. As with the SANS, each subscale also includes a global rating scale.

SANS and SAPS global and subscales scores are shown in Table 1.

### Circles-Lines Coupling Task

**Figure 1:**
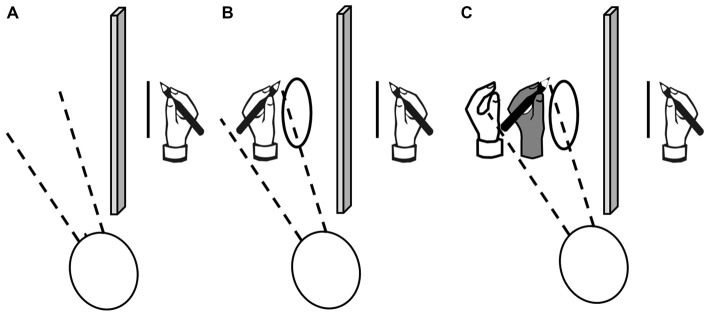
**Graphical representation of the experimental paradigm.** From left to right: **(A)** baseline condition where patients performed lines with their right hand (UL); **(B)** bimanual circles-lines condition where patients performed line with their right hand and simultaneously performed circles with their left hand (BCL); **(C)** observation condition where patients performed lines with their right hand while simultaneously watching circles performed by an alien hand (in dark gray in the figure) in their right egocentric peri-personal space (OC). To be noted that in all three conditions, participants had to watch on their left peri-personal egocentric space and vision of their right hand was precluded by a paper separator.

Participants were asked to perform unimanual and bimanual movements, continuously drawing vertical lines and/or circles without interruptions for 12 s for each trial. The subjects sat on a chair with both hands lying on the table on which a tablet PC was positioned to the right of the subject’s sagittal midline. The view of the right hand was blocked with a cardboard panel and subjects were explicitly requested to focalize the attention on the left side. In all conditions, the right hand trajectories were automatically recorded by the tablet PC whereas, in bimanual or observation conditions (see below), the left hand had to draw on a sheet of paper. In the alien conditions, also an alien hand (the examiner’s one) was on the table. It was a left alien hand aligned with the patient’s left shoulder, that is in a congruent position with respect to the patient’s trunk midline, between the patient’s body and the patient’s left hand (observation circle condition). A white sheet was used to cover the patients’ and the examiner’s arms. It is worth noting that in all conditions the subject’s left hand was visible on the table. As shown in Figure 1, in the experimental design, there were one baseline condition and two experimental conditions:

1. Baseline Unimanual Lines (UL): participants were asked to draw lines with the unseen right hand;
2. Bimanual Circles and Lines (BCL): participants were asked to simultaneously draw lines with the unseen right hand and circles with the left hand;
3. Observation Circles (OC): participants were asked to draw lines with the unseen right hand and to observe the alien left hand drawing circles.

In the experimental procedure there were 10 trials for each condition, for a total of 30 trials. We used the following balanced sequence: UL; BCL; OC; OC; BCL; UL.

### Instrumented Analysis of Bimanual Coupling During Drawing

An Ovalization Index (OI) was calculated, according to previous studies (see Garbarini et al., 2012, 2013a, 2015b; Piedimonte et al., 2014), as the standard deviation (SD) of the right-hand trajectories in relation to an absolute vertical line. For a throughout description of the steps involved in calculating the OI refer to Garbarini et al. (2012). Briefly, OI index ranges between a value of 0 for straight trajectories without any sign of ovalization and a value of 100 for circular trajectories. The amount of interference of the left (own or alien) hand executing circles on the right hand executing lines is shown as an increase of the OI (bimanual coupling/interference effect) compared to the baseline UL.

### Ownership and Agency “Online” Evaluation in Patients With Schizophrenia

In order to evaluate the patients’ sense of body-ownership and sense of agency, during the OC condition in which the examiner’s hand drew circles, at the end of each block of five trials, an ownership/agency questionnaire was administered. Patients were asked to answer using a 7-point Likert scale from −3 (i.e., I don’t agree at all) to 3 (i.e., I totally agree) if they were in agreement/disagreement with the following statements:

1. “*I felt as if the alien hand was my hand*” (Body-ownership statement)
2. “*The alien hand moved just like I wanted it to, as if it was obeying my will*” (Agency statement).

These statements were adopted from existing questionnaires used in traditional rubber hand illusion experiments (Kalckert and Ehrsson, 2012).

### Statistical Analysis

For each participant, the OI mean value for the experimental BCL and OC conditions was expressed as percentage of the baseline UL condition (BCL% = [OI_BCL_/OI_UL_]*100; OC% = [OI_OC_/OI_UL_]*100). The obtained values were analyzed by using unpaired *t*-tests (two tailed), to compare patients and controls during both BCL and OC conditions. Furthermore, in patients with schizophrenia, unpaired *t-tests* (two tailed) were used to compare acute and stable patients in both BCL and OC conditions. A paired *t-test* (two tailed) was used to compare the patients’ performance in BCL and OC conditions. Finally, in patients, we estimated the correlations (Pearson’s r) between the BCL/OC% of the baseline, SAPS and SANS global and subscales scores and the ratings obtained from the ownership/agency questionnaire.

## Results

### Comparison Between Patients and Controls During the Bimanual Task

**Figure 2:**
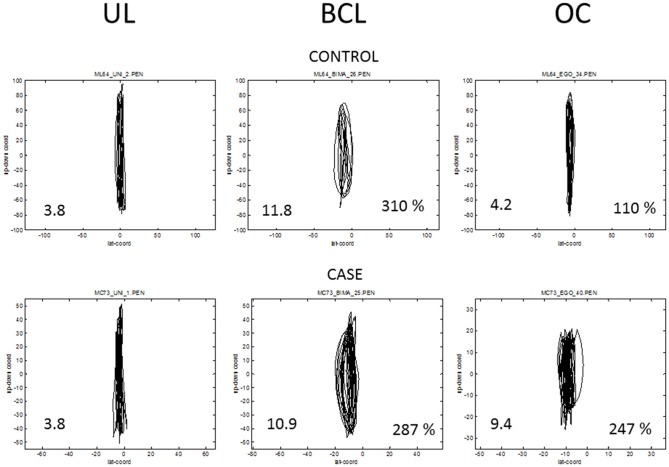
**Examples of the right hand trajectories in schizophrenic case (below) and a control subject (above) in Unimanual Lines (UL), Bimanual Circles and Lines (BCL) and Observation Circles (OC) conditions.** The Ovalization Index (OI) is reported inside each of the plots, at bottom left, and in BCL and OC conditions it is reported, at bottom right, also the percent ratio relative to the UL condition. Both control and case show the expected ovalization in the BCL condition, but, when considering the Observative condition, only the case shows a residual ovalization.

When controls and schizophrenics were asked to perform the bimanual circles-lines task, the expected coupling effect was evident as a clear ovalization of the line trajectories, performed by the right hand, due to the interference of the circles simultaneously performed by the left hand. See examples in Figure 2.

**Figure 3:**
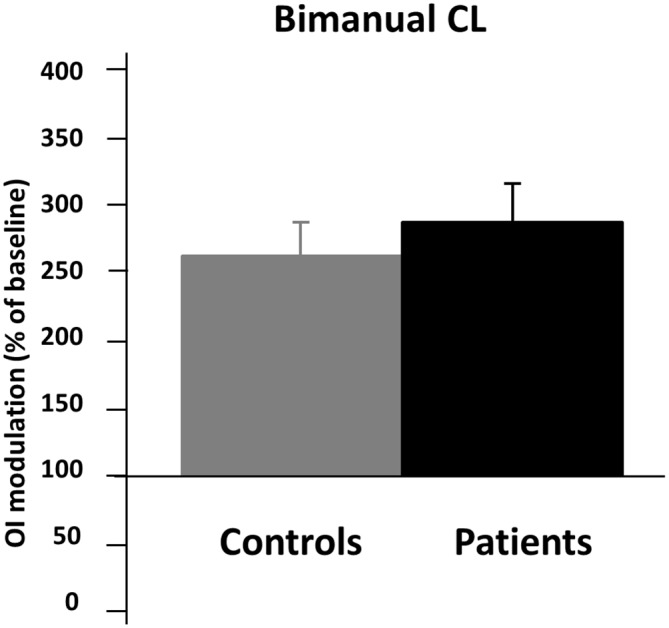
**Results from the comparison between schizophrenics and controls during BCL condition.** On the *y* axis, the OI of the BCL condition (bimanual circles and lines expressed as % of the baseline condition (unimanual lines condition). Black bars represent patients’ conditions while gray bars represent controls conditions. Error bars represent standard errors of the mean (SEMs).

The statistical comparison between patients and controls during BCL condition indicates no significant difference between the two groups (BCL% of baseline mean ± SD; Controls: 265.09 ± 102.38; Patients: 287.06 ± 147.95; *T* = −0.54, *p* = 0.58, Unpaired *t*-test, two tailed; see Figure 3).

When comparing acute and stable patients, no difference was found during BCL condition (BCL% of baseline mean ± SD; Acute Patients: 257.36 ± 118.85; Stable Patients: 272.82 ± 89.1; *T* = −0.32, *p* = 0.74, Unpaired *t*-test, two tailed).

### Comparison Between Patients and Controls During the Observation Task

During the observation version of the circles-lines task, a stronger coupling effect was evident in patients than in controls, as a greater ovalization of the line trajectories, performed by the right hand, due to the interference of the circles performed by the examiner’s left hand. See examples in Figure 2.

**Figure 4:**
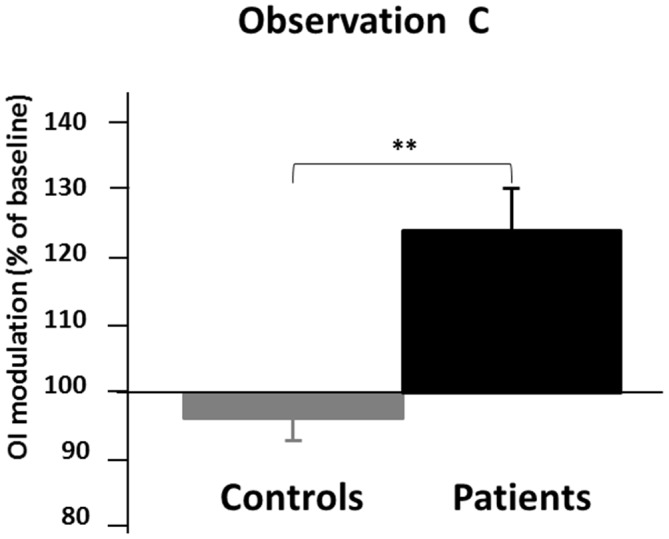
**Results from the comparison between schizophrenics and controls during OC condition.** On the *y* axis, the OI of the OC condition (observation of circles performed by an alien hand while performing lines) expressed as % of the baseline condition (UL condition). Black bars represent patients’ conditions while gray bars represent controls conditions. Error bars represent standard errors of the mean (SEMs). **p < 0.01.

The statistical comparison between patients and controls during OC condition indicates significant difference between the two groups (OC% of baseline mean ± SD; Controls: 97.34 ± 16.94; Patients: 113.82 ± 25.46; *T* = −2.75, *p* < 0.01, Unpaired *t*-test, two tailed; see Figure 4).

When comparing acute and stable patients, no difference was found during OC condition (OC% of baseline mean ± SD; Acute Patients: 118.21 ± 28.04; Stable Patients: 109.43 ± 23.22; *T* = 0.76, *p* = 0.45, Unpaired *t*-test, two tailed).

In both patients and controls, when comparing the obtained values in BCL and OC conditions, a significant difference was found (*p* < 0.001 for each comparison; Paired *t*-test, two tailed).

### Correlations Results in Patients

**Figure 5:**
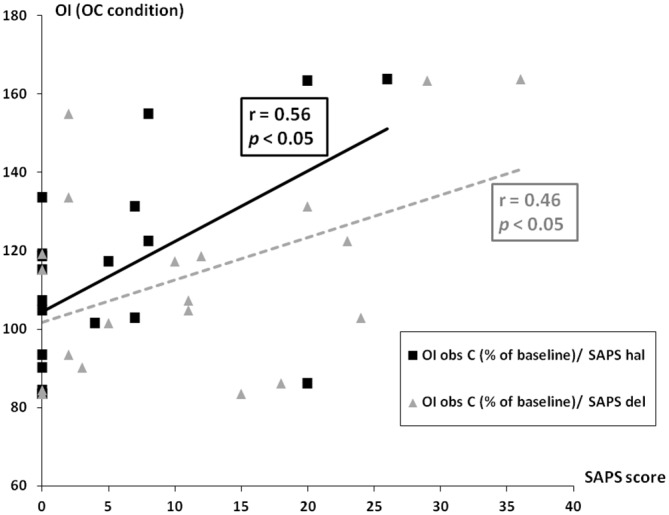
**Correlation between OC and scale for the assessment of positive symptoms (SAPS) global and subscales scores.** On the *y* axis, the OI of the OC condition (observation of circles performed by an alien hand while performing lines) expressed as % of the baseline condition (UL condition). On the *x* axis, SAPS scores. Black squares represent comparison of Observation C with SAPS hallucinations subscale scores (SAPS hal) while gray triangles represent comparison of Observation C with SAPS delusion subscale scores (SAPS del).

**Figure 6:**
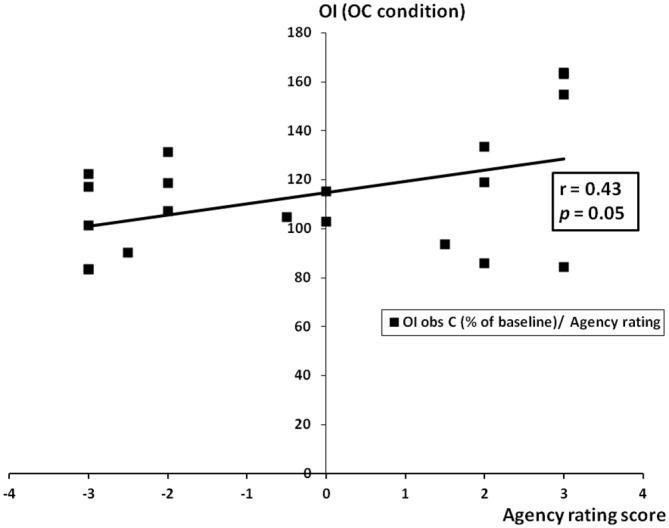
**Correlation between OC condition and agency rating scores.** On the *y* axis, the OI of the OC condition (observation of circles performed by an alien hand while performing lines) expressed as % of the baseline condition (UL condition). On the *x* axis, the agency rating scores.

**Figure 7:**
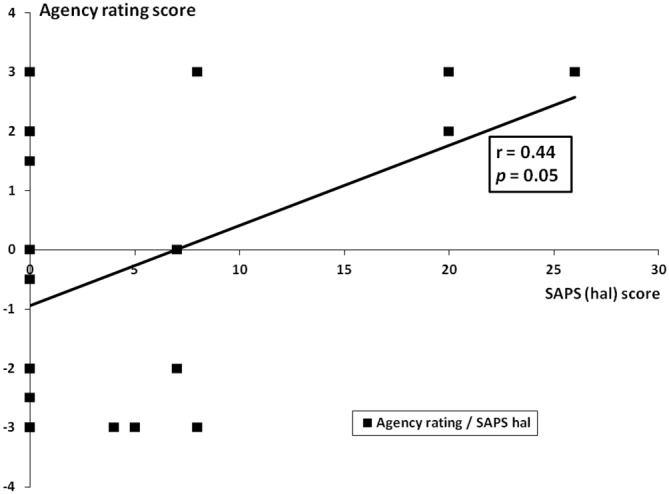
**Correlation between agency rating and SAPS hallucinations subscale scores.** On the *y* axis, the agency rating scores while on the *x* axis, the SAPS hallucinations subscale scores (SAPS hal).

We examined whether, in patients with schizophrenia, a relation exists between the values obtained in BCL and OC conditions and both SAPS and SANS global and subscales scores and the Ownership/Agency ratings. The significant differences between acute and stable patients in SAPS and SANS global and subscales scores are reported in Table 1. With respect to the Ownership/Agency ratings, no significant differences were found between acute and stable patients (Ownership ratings ± SD; Acute Patients: −1.95 ± 2.14; Stable Patients: −1.05 ± 2.2; Agency ratings ± SD; Acute Patients: −0.55 ± 2.45; Stable Patients: 0.1 ± 2.5; *p* > 0.3 for each comparison; Unpaired *t*-test, two tailed). Correlation analysis showed that the OC value was significantly related with SAPS global score and with hallucinations and delusions subscales scores (SAPS global score: *r* = 0.43, *p* = 0.03; SAPS Hallucinations subscale score: *r* = 0.56, *p* = 0.01; SAPS Delusions subscale score *r* = 0.46, *p* = 0.04; see Figure 5). Furthermore, OC values significantly correlated with the ratings given by the patients at the Agency questionnaire: “*The alien hand moved just like I wanted it to, as if it was obeying my will”* (*r* = 0.43, *p* = 0.05; see Figure 6). Finally, a significant correlation existed between SAPS Hallucination subscale scores and the Agency ratings (*r* = 0.44, *p* = 0.05; see Figure 7). There were no significant correlation with SANS global and subscales scores and with the ratings at the Ownership questionnaire.

## Discussion

The present study is related to one of the most important component of the human self-awareness; i.e., the sense of agency, that is the sense of being the agent of a willed action (Gallagher, 2000; Jeannerod, 2003). In particular, we sought for evidence that, in schizophrenia, an excessive sense of agency, for externally generated actions, affects the patients’ motor parameters during a bimanual circles-lines task.

The bimanual interference is a highly reproducible effect, always present across different ages (Piedimonte et al., 2014), induced by different tasks, in both spatial (e.g., Franz et al., 1991) and temporal (e.g., Pia et al., 2013) domain. The neural mechanism subserving this phenomenon is supposed to be the default coupling of homologous muscles, promoted by the neural crosstalk. Converging neuroimaging evidence show that, during non-congruent bimanual movements (as those performed in the circles-lines task), the pre-supplementary motor area (pre-SMA) exerts an inhibitory function on this default coupling, thus allowing the execution of non-congruent bimanual movements (e.g., Sadato et al., 1997; Wenderoth et al., 2004; Garbarini et al., 2013a).

In the present study, patients and controls showed a comparable interference effect during the “classical” bimanual version of the circles-lines task (i.e., no difference was found between patients and controls for the OI enhancement in BCL condition respect to UL condition). This suggest that, at least in our schizophrenics sample, no motor abnormalities, specific for bimanual tasks, can be described (neither in acute nor in stable patients, showing a comparable interference effect in BCL condition). On the contrary, in the observation version of the task, the results showed a significant difference between schizophrenics and controls. In particular, in schizophrenics, respect to controls, we found a greater interference effect of the examiner’s left hand movements, performing circles, on the actual movements of the patients’ right hand, performing lines. No significant difference in OC condition was found between acute and stable patients, suggesting that the effect we found does not depend on the phase of the illness. Crucially, in patients, the interference enhancement was related to the strength of the sense of agency reported during the task (the patients’ rating at the Agency statement: “*The alien hand moved just like I wanted it to, as if it was obeying my will*”).

Human’s sense of agency can be measured with at least two different paradigms. An explicit paradigm consists in asking participants to verbally indicate how much they sense a feeling of control and of authorship over an action. An example of implicit paradigm to evaluate the sense of agency is the “intentional binding” effect in subjective time experience during an intentional action (Haggard and Eimer, 1999; Haggard et al., 2002). As before mentioned (see “Introduction” Section), both explicit and implicit paradigms have been employed in schizophrenia. Daprati et al. (1997), asked the patients to execute simple hand movements (without visual control) while showing, in real time, a video of either own hand or an alien hand, executing the same or a different movement. The patients’ sense of agency was explicitly evaluated by asking the patients if they saw on the screen their own hand performing the movement. They found that, hallucinating and deluded patients with schizophrenia were more impaired in discriminating their own hand from the alien one than the non-hallucinating ones, and tended to misattribute the alien hand to themselves. Franck et al. (2001) asked the patients to hold a joystick and to perform discrete movements in different directions while showing them the image of a virtual right hand holding a joystick. Angular biases and temporal delays were randomly introduced in some trials, such that the movement of the virtual hand departed from the movement executed by the subjects. The patients’ sense of agency was explicitly evaluated by asking the patients whether the movement they saw was their own. They found that, compared with normal subjects, deluded schizophrenics made significantly more recognition errors. On the other side, studies employing the intentional binding, as an implicit measure of the sense of agency, showed that patients feel both forward (when the external events follow their intentional actions; Haggard et al., 2003; Voss et al., 2010) and backward (when the external events precede their intentional actions; Maeda et al., 2012) exaggerated causal efficacy (i.e., an hyper-sense of agency) in the temporal event sequence during the intentional action. In the present study, we investigated the sense of agency by jointly employing both explicit (Agency rating) and implicit (OI value in OC condition) measures, also showing a significant correlation between them.

It is interesting to note that in the reviewed papers, employing either explicit or implicit measure of the sense of agency, a correlation with the positive symptoms, in particular hallucinations and delusions, has been reported as crucial in order for the effects of interest to occur (Daprati et al., 1997; Franck et al., 2001; Voss et al., 2010; Maeda et al., 2012). Also in our schizophrenics’ sample, a significant correlation was found between positive symptoms (in particular hallucinations and delusions as identified by SAPS hallucinations and delusions subscales scores) and both the explicit and the implicit measure of the sense of agency: the higher the positive symptoms, the higher both the patients’ agency rating and the OI value in OC condition. Positive symptoms could be defined as an excess or a distortion of normal functions (thoughts, emotions or behavior). Within the positive symptomatology, the patients may attribute their own action or thought to an external source, or they may experience the reverse as well; i.e., they may perceive the others’ action as a consequence of their own intentions and they may have the strong tendency to “bind” external events to their actions and feel they cause all external phenomena around them. According to previous studies (Daprati et al., 1997; Franck et al., 2001; Voss et al., 2010; Maeda et al., 2012), our data provide experimental evidence supporting the hypothesis that positive symptoms seem to be relevant to a dysfunction of the awareness of action in schizophrenia.

Positive symptoms are typical of the acute stage of the disease but can be present throughout the different phases of the disorder. In our schizophrenics’ sample, although the positive symptomatology was stronger in acute than in stable patients, the correlations with positive symptoms (hallucinations and delusions) were still significant also when the phases of the illness (acute/stable) was controlled for.

The sense of agency has to be distinguished from a sense of body-ownership, which is the perceptual condition that allows the subject to ascribe body parts to oneself (Tsakiris et al., 2007). The sense of agency and the sense of body-ownership are fundamental components of self-awareness and they interact in determining a conscious feeling of the self as an acting body. Previous studies showed that patients with schizophrenia have a more flexible body representation and weakened sense of self. Thakkar et al. (2011) demonstrated that watching a rubber hand being stroked, while one’s unseen hand is stroked synchronously (i.e., the phenomenon known as the Rubber Hand Illusion), can lead to qualitative (the reported sense of ownership over the rubber hand) and quantitative (a shift in perceived position of the real hand and a limb-specific drop in stimulated hand temperature) stronger effects in schizophrenics compared to controls. Ferri et al. (2012) investigated the schizophrenics’ performance during an implicit self-recognition task (visual matching), employing both participant’s own and others’ body-effectors. Schizophrenics did not show the typical self-advantage described in healthy subjects (i.e., when the visual matching task involves the own body-effectors, the healthy subjects’ accuracy is greater than when it involves the others’ body-effectors). In our study, we did not find any significant correlation with the patients’ rating at the Body-ownership statement: “*I felt as if the alien hand was my hand*”. This suggests that the interference effect we found in OC conditions can be better explained by the sense of agency (showing a significant correlation with the OI parameter) than by the sense of ownership the patients experienced during the task for the examiner’s hand drawing circles.

In this balance between agency and ownership, the difference between the results found in psychiatric and neurological patients can be addressed. In schizophrenics, tested here, the effect we found in the observation version of the circles-lines task was not comparable to that found in the bimanual version of the task (i.e., a significantly greater effect was found in BCL condition then in OC condition). On the contrary, in brain-damaged patients with pathological embodiment the interference effect found in the observation task was very similar to that found in healthy subjects actually performing bimanual Circles-Lines task (i.e., no difference between BCL condition and OC condition was found; see “Introduction” Section and for a full description Garbarini et al., 2013b). During the observation task, neurological patients with pathological embodiment showed a profoundly altered sense of body ownership, claiming that the examiner’s hand drawing circles was their own hand. This primary body-ownership deficit, in turn, affected the patients’ motor awareness and sense of agency (i.e., the patients ascribed the examiner’s movement, drawing circles, to themselves). On the contrary, schizophrenics were always conscious that, in the experimental setting, the hand drawing circles was not their own, although hallucinated and deluded patients reported to feel *as if* “the alien hand moved just like they wanted it to, as if it was obeying their will”. However, what these data tell us is that, both in neurological and in psychiatric patients, the delusional beliefs are not mere verbal confabulations, but instead have objective consequences on the patients’ motor performance. In particular, in schizophrenics, we found that the greater the patients’ agreement over the agency statement (“*The alien hand moved just like I wanted it to, as if it was obeying my will*”), the greater the modulation of the patients’ motor performance. We can speculate that, in schizophrenics, the delusion of agency can automatically trigger the intention-programming processes for the own hand, as if the alien hand was actually “obeying to the patients will”, so that, when the examiner’s hand drew circles, the lines drawn by the patients’ intact hand become ovalized.

In normal conditions, the feeling of being the agent of a willed action is so self-evident and obvious that people think there is nothing to explain about it. In fact, the study of pathological conditions can reveal that the awareness of willed action is build up in a complex way and that its coherence can be severely and unexpectedly affected. The data discussed here show that, in schizophrenia, an excessive sense of agency, for externally generated actions, affects the patients’ motor parameters during a bimanual circles-lines task. Compared to controls, schizophrenics showed a significantly greater interference effect of the examiners’ hand drawing circles on the own hand drawing lines. This interference enhancement was related to the strength of positive symptoms (SAPS global score and with hallucinations and delusions subscales scores) and to the sense of agency reported by the patients during the task (Agency rating). These findings suggest that an altered sense of agency, as shown by schizophrenics, can induce objective consequences on the motor system.

## Author Contributions

FG, LP, MS, AP and PR, designed the study; MS and PR selected patients; MR carried out the software to acquire data; AM acquired data; FG, MR and AM analyzed data; AP carried out the figures; MS and AM carried out the tables; FG, LP, MS and AM wrote the manuscript.

## Funding

This work has been funded by MIUR-SIR 2014 grant (RBSI146V1D) to FG and by MINSAN Ricerca Corrente 2015 (C15.1) to MR.

## Conflict of Interest Statement

The authors declare that the research was conducted in the absence of any commercial or financial relationships that could be construed as a potential conflict of interest.
